# Liquid-liquid phase separation of membrane-less condensates: from biogenesis to function

**DOI:** 10.3389/fcell.2025.1600430

**Published:** 2025-05-14

**Authors:** Huanyu Guan, Hui Wang, Xin Cai, Jiabo Wang, Zhixin Chai, Jikun Wang, Haibo Wang, Ming Zhang, Zhijuan Wu, Jiangjiang Zhu, Jincheng Zhong, Binglin Yue

**Affiliations:** Key Laboratory of Qinghai-Tibetan Plateau Animal Genetic Resource Reservation and Utilization, Sichuan Province and Ministry of Education, Southwest Minzu University, Chengdu, China

**Keywords:** MLC, LLPS, IDR, lncRNA, skeletal myogenesis

## Abstract

Membrane-less condensates (MLCs) are highly concentrated non-membrane-bounded structures in mammalian cells, comprising heterogeneous mixtures of proteins and/or nucleic acids. As dynamic compartments, MLCs can rapidly exchange components with the cellular environment, and their properties are easily altered in response to environmental signals, thus implicating that they can mediate numerous critical biological functions. A basic understanding of these condensates’ formation, function, and underlying biomolecular driving forces has been obtained in recent years. For example, MLCs form through a liquid-liquid phase separation (LLPS) phenomenon similar to polymer condensation, which is primarily maintained via multivalent interactions of multi-domain proteins or proteins harboring intrinsically disordered regions (IDRs) as well as RNAs with binding sites. Moreover, an accumulating body of research indicates that MLCs are pathophysiologically relevant and involved in gene expression regulation and cellular stress responses. Here, we review the emerging field and explore what is currently known about the varied progress in LLPS of MLCs and how their features affect various cellular process, focusing on RNAs, including in skeletal myogenesis.

## 1 Introduction

Each mammalian cell contains many molecules with different physicochemical properties, which can all interact somewhat ordered. An evolutionary conserved way to achieve such control is the creation of cellular compartmentalization for the efficient organization of molecules, biochemical reactions, and signals (Feric et al., 2016). For instance, canonical organelles such as the endoplasmic reticulum, Golgi apparatus, mitochondria, and secretory vesicles are membrane-bound structures that provide the physical separation required for unique biochemical processes to occur within a cell (Helle et al., 2013). Another type of compartment is termed membrane-less organelles (MLOs); however, they maintain a well-defined composition, structure, and function without the need for membranes. MLOs are defined as biomolecular condensates sized approximately 0.2–2 μm, and some more minor and transient biomolecular condensates, such as transcriptional condensates, are not usually described as MLOs. Hence, we collectively term them MLCs in this review (Hirose et al., 2023; Dl, 2006). For a long time, numerous MLCs within the nucleus and the cytoplasm, such as nucleolus, P granules, peckles, centrosomes, cytosolic processing bodies (P-bodies), and stress granules (SGs), are successively discovered, however, due to technical limitations connected to biochemical isolation, MLCs study has mainly been restricted to mere observation (Handwerger et al., 2005). With recent advances in experimental methods combining mass spectrometry, *in vivo* labeling techniques, and high-resolution microscopy imaging, it is clear that phase separation is a major driving force for the formation of cellular MLCs.

**FIGURE 1:**
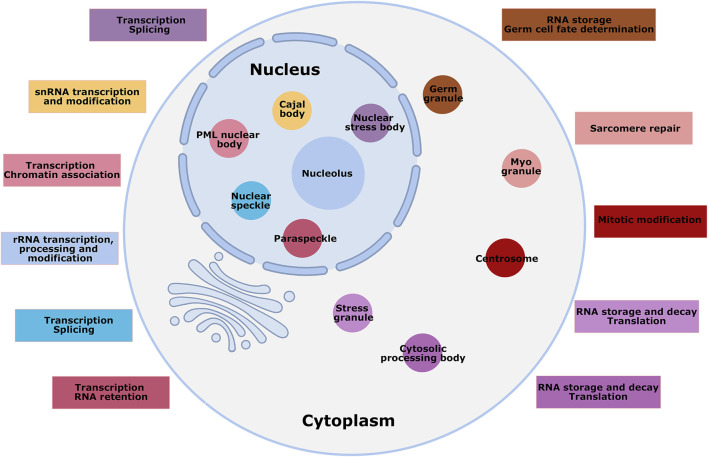
Overview of MLCs of mammalian cells. Numerous MLCs are shown in the nucleus and cytoplasm of mammalian cells, not to scale, with a brief description of their functions. Some MLCs are present only in specified cell types; for instance, germ granules and myo-granules are specific to germ and muscle cells. Different colors mark the MLCs and their functional description.

Phase separation is a well-understood physiochemical phenomenon that indicates generating multiple distinct phases out of homogeneous solutions. In mammalian cells, phase separation (predominantly LLPS) leads to the concentration of specific biological macromolecules, which can act as membrane-less compartments that separate in space and time to perform different functions (Banani et al., 2017) (Figure 1). Under LLPS, some proteins form liquid-like condensates, which can flow and fuse or even deform when pressure is applied, and these condensates sometimes change into solid-like forms (liquid-solid phase separation) in pathological scenarios and are sometimes disassembled in physiological scenarios (Bose et al., 2022). Note that other biological macromolecules, such as DNA, RNA, and even glycogen, are extensively involved in this process (Liu et al., 2021; Zhao et al., 2022; Langdon and Gladfelter, 2018). The mixed biomolecular interaction modes within the intracellular environment yield dynamic MLCs facilitating various biological functions.

Mammalian skeletal myogenesis is a complex process that allows precise control of myogenic cells’ proliferation, differentiation, and fusion to form multinucleated, contractile, and functional muscle fibers (Bentzinger et al., 2012; Buckingham et al., 2003). Over the years, these processes have been intensively studied using the skeletal muscle developing models *in vitro* and *in vivo* and uncovered a complex cellular intrinsic network during mammalian skeletal myogenesis containing transcription factors, translation factors, extracellular matrix, metabolites, and mechanosensors; however, it remains unclear how the properties of MLCs exactly contribute to these effects (Bryson-Richardson and Currie, 2008; Knight and Kothary, 2011; Wu and Yue, 2024). In the review, we seek to provide an overview of the recent advances related to the biology, dynamics, and various functions of LLPS-driven MLCs and their relevance in skeletal myogenesis, providing insights into skeletal muscle pathophysiology.

## 2 The molecular mechanisms of LLPS

**FIGURE 2:**
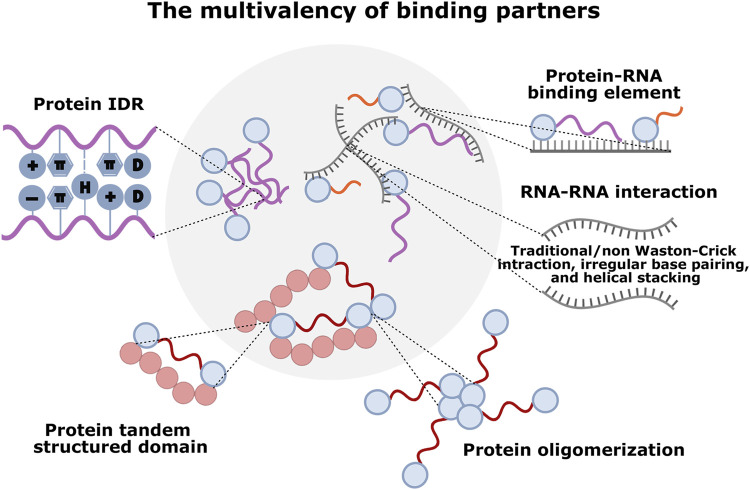
The multivalency of different molecular components driving LLPS in condensate formation. Various multivalent interactions sustain phase-separated MLCs: protein-protein interaction mediated by IDRs (cation–π, π–π stacking, charge-charge, dipole-dipole, and hydrogen bonding interactions), tandem structured domains, or oligomerization; RNA-RNA interaction mediated by self-assemblies (irregular base-pairing, helical stacking, and traditional/non-Watson-Crick interactions); protein-RNA interaction mediated by RNA-binding domains (RBDs) and/or IDRs with RNA element.

LLPS is a process that influences the properties of fluids with different densities and governs their behavior. It arises from the supersaturation of molecules: molecules will be miscible in solution until they reach the threshold concentration (Banani et al., 2017). By weak, multivalent interactions between macromolecules, LLPS can drive the selective condensation of different molecules into each membrane-less compartment, assembling dynamic structures with liquid-like physical properties in biological systems. The availability of multiple binding sites in a molecule or a complex is termed multivalency, which has numerous manifestations: protein multiple modular interaction domains, IDRs of proteins, and multiple protein-binding elements within nucleic acids (Gao et al., 2022; Li et al., 2012). The multivalency of binding partners allows the local dynamic engagement to the overall mutual affinity (Figure 2).

### 2.1 Protein multiple modular interaction domains

Proteins are typically modular and composed of multiple domains. Some consist of multiple copies of the same domain type, called protein tandem structured domains (Figure 2). These multivalent domains are usually involved in protein-ligand interactions, and the ligands could be protein domains, peptide motifs, RNA/DNA motifs, and sugar residues within polysaccharides or lipid moieties (Zhao et al., 2022; Gao et al., 2022). Moreover, the multivalency of protein allows its simultaneous dynamic engagement with multiple binding partners, which increases the overall mutual affinity to enable phase separation at lower concentrations (Li et al., 2012). A classic example was the phase transition in assembling the protein nephrin and its partner Nck and neural Wiskott-Aldrich Syndrome protein (N-WASP) in the actin-regulatory signaling pathway. In the nephrin/Nck/NWASP system, nephrin contains three phosphotyrosine (pTyr) motifs, which bind to the SH2 domains in Nck, while Nck also contains three SH3 domains, which interact with proline-rich motifs (PRMs) in N-WASP. Governed by the degree of nephrin phosphorylation, the assembly forms phase-separated condensates suspended in solution (Li et al., 2012). Apart from the signaling system, MLCs such as postsynaptic densities (PSDs), p62 bodies, and nucleolar protein 1 (NPM1) were also shown to perform phase separation through protein multiple modular interaction domains. PSDs are semienclosed MLCs beneath postsynaptic membranes that coordinate synaptic structure and functions, in which the most abundant PSD protein, SynGAP, forms a parallel coiled-coil trimer and binds to PDZ domains of PSD-95 through C-terminal PDZ binding motif, inducing the formation of LLPS both *in vitro* and in living cells (Zeng et al., 2016). p62 bodies, the MLCs assembling ubiquitin-tagged misfolded proteins for selective autophagy, whose phase separation depends on the N-terminal PB1 domain and a C-terminal ubiquitin-associated (UBA) domain of p62. Mechanistically, p62 oligomerizes via its PB1 domain and binds to the polyubiquitin chain via its UBA domain (Sun et al., 2018). An analogous system controlling nucleolus organization in eukaryotic cells, a nucleolar chaperone NPM1 assembles into pentamers via its N-terminal domain oligomerization and engages in weak interactions with proteins that contain arginine-rich linear motifs (R-motifs) via its conserved acidic tracts. Moreover, NPM1 can also bind to potentially multivalent nucleic acids via its folded nucleic acid binding domains, which is required for phase separation of NPM1 with other nucleolar components *in vitro* and in cells that mediate ribosome biogenesis and nucleolar stress responses (Mitrea et al., 2016). Modular multivalent proteins often undergo phase separation in the presence of their binding partners; however, molecular assembly does not necessarily lead to phase separation, even if it is usually coupled. For example, a spontaneous assembly is driven by specific recognition between the tandem repeated CC43 WW domain and PRMs, respectively derived from protein polymers C7 and P9, resulting in a single, homogeneous hydrogel formation (Mulyasasmita et al., 2011).

### 2.2 IDRs

Unlike folded domains that adopt persistent secondary and tertiary structures, IDRs are protein segments with less solid structure and heterogeneous and dynamic conformation. IDRs usually contain repeated sequence elements based on their biased amino-acid distribution, which is essential for targeting partners and LLPS *in vitro* and living cells (Wang and Zhang, 2019). Not all IDRs can promote LLPS, and phase-separating IDRs often share some sequence features. A subset of IDRs, the low-complexity domains (LCDs) have low sequence complexity and exhibit compositional bias toward a limited set of amino acids, primarily including aromatic residues (Phe and Tyr), charged residues (Lys, Arg, Asp, and Glu), and uncharged polar residues (Gly, Ser, Asn, and Gln), which form a variety of side-chain interaction types (cation–π, π–π stacking, charge-charge, dipole-dipole, and hydrogen bonding interactions) to facilitate phase separation (Hirose et al., 2023) (Figure 2). For instance, cytoplasmic messenger ribonucleoprotein (mRNP) granules, such as germ granules, P-bodies, and SGs, are LCD-driven MLCs involved in mRNA metabolism and homeostasis (Buchan, 2014; Mitrea and Kriwacki, 2016). In the cases of assembly of RNA helicase DDX4 in germ granules, formation is facilitated by cation–π and π–π stacking interactions between the repeated dipeptides Arg-Gly/Gly-Arg and Phe-Gly/Gly-Phe in Ddx4 N-terminus, methylating arginine residues or Phe to Ala mutations all inhibit phase separation (Nott et al., 2015). Electrostatic interactions between clusters of opposing charges in Ddx4 suggest charge-charge types driving forces for phase separation (Nott et al., 2015). Similarly, through electrostatically driven interactions, the Arg/Gly-rich (RGG) domain of *Caenorhabditis elegans* protein LAF-1 facilitates self-assembly, phase-separating into P-granule-like condensates *in vitro* (Elbaum-Garfinkle et al., 2015). Nuclear pore complex formation also requires Phe-Gly repeats, which can mediate π–π stacking for all exchanges between nuclei and cytoplasm (Frey et al., 2006). Furthermore, sequences enriched in uncharged polar residues serve as a driving force for phase separation through dipolar interactions of their side chains. Several proteins including FUS, hnRNPA1/2, TDP-43, and Lsm4p consist of low-complexity sequences termed prion-like domains(PLDs), whose sequences often encompass multiple short motifs such as Ser/Tyr/Gly/Gln (SYGQ), Gln/Asn (Q/N)-rich regions, and the net attraction between peptide dipoles makes them phase separation at low micromolar concentrations (King et al., 2012; Reijns et al., 2008; Decker et al., 2007; Molliex et al., 2015). In addition to side-chain interactions, the capacity for main-chain hydrogen bonding also contributes to the formation of labile cross-β structure, promoting self-assembly and phase separation of LCDs, as in FUS, hnRNPA1/2, and TDP-43 (Murray et al., 2017; Xiang et al., 2015; Zhou et al., 2022). In addition, there seems to be a hierarchical interplay among these different interaction types: electrostatic force between charged residues promotes long-range interactions, and short-range interactions depend on directional interactions of dipoles (Gly, Gln, Asn, Ser) or positive charges (Arg) with aromatic groups (Phe and Tyr) (Bianchi et al., 2020). Thus, although various amino-acid compositions greatly influence MLC formation, which IDR sequences are most conducive to LLPS remains unclear.

### 2.3 Multiple protein-binding elements within RNA

RNA is another essential participant in LLPS, whose many properties can facilitate its phase separation with RNA-binding proteins (RBPs) and negative charge. It is a crucial RNA property that binds proteins together to promote phase separation owing to charge effects (Aumiller et al., 2016). The multivalency of RNA partly derives from its sequences, and specific protein-binding elements within RNAs are often found in multiple copies interspersed across the sequence, which are recognized by the RNA-binding domains (RBDs) on RBPs (Cereda et al., 2014). A classic example is that multivalent RNA-recognition motifs (RRMs), the highly folded RBD, can bind multiple short and degenerate RNA motifs to increase the overall mutual affinity (Maris et al., 2005). Whi3, an RBP with a long polyQ tract adjacent to an RRM, CLN3, and BNI1 mRNA binding via its RRM are required for Whi3 phase separation and drive Whi3 to form condensates with distinct biophysical properties (Zhang et al., 2015). In addition to RRM, RNAs are bound via a series of IDRs on the RBP to drive phase separation. For example, the disordered N-terminal RGG domain of LAF-1 interacting with RNA is essential for LLPS. RNA can decrease the viscosity of protein condensates and increase internal molecular dynamics (Elbaum-Garfinkle et al., 2015).

Additionally, the multivalent base-pairing propensity of an RNA can also lead to their phase separation without requiring protein components. It has been suggested that RNA-RNA interactions contribute to both SGs assembly and targeting of RNAs to SGs (Van Treeck et al., 2018). These interactions contributing to RNA self-assemblies could be traditional/non-Watson-Crick (WC) interactions, irregular base-pairing, or helical stacking (Jain and Vale, 2017; Zanchetta et al., 2008; Leontis et al., 2002) (Figure 2). Especially for non-WC interactions, more vital interaction between RNA with ALS/FTD-associated GC repeats found in the C9orf72 locus can lead to their phase separation in RNA that can form stable G-quadruplex structures (Jain and Vale, 2017). vitro and in cells, which is dependent upon the number of polys Moreover, the other three homopolymers can self-assemble, with polyC forming slow relaxing condensates, polyA forming asymmetrical slower relaxing condensates, and polyU forming rapidly relaxing condensates (Aumiller et al., 2016; Van Treeck et al., 2018). Beyond binding to protein and RNA, the features of RNA that favor LLPS include RNA length, single-strandedness, or other characteristics. For example, RNA enrichment in SGs appears to be related to RNA length rather than specific sequence motifs, and a recent study revealed a strong correlation between mRNA length and the G3BP1’s ability to drive LLPS in SGs (Yang et al., 2020). In the same case, single-stranded RNAs, but not double-stranded RNAs, are required to trigger LLPS with G3BP1, suggesting that G3BP1 binds RNA in cells without clear sequence preference (Yang et al., 2020). Therefore, various physiochemical properties of RNA support their extensive association with phase-separated condensates; however, the deep understanding of these properties still needs to be improved.

### 2.4 The synergy between multivalent components

Individual phase-separated MLCs usually contain hundreds of different molecular components, such as SGs and P-bodies, primarily accumulations of hundreds of proteins and thousands of RNA transcripts (Khong et al., 2017; Markmiller et al., 2018; Hubstenberger et al., 2017). An analysis of these components in MLCs suggested that only a few appear essential for structural integrity, termed scaffolds, drive phase separation through multivalent interactions. Depletion of scaffolds may decrease the size and/or number of the structures in a cell. However, other components called clients are often recruited by scaffolds via low-valency interactions but are not required for condensate formation (Banani et al., 2016). Many proteins that facilitate assembly contain folded domains and IDRs, which can mediate specific interactions to form scaffolds for the assembly of MLCs and provide organizational clients with the ability to recruit additional components through weaker, dynamic interactions (Mitrea and Kriwacki, 2016). Promyelocytic leukemia protein (PML) is an example of a scaffold, the only protein essential for PML nuclear body formation. In contrast, for clients such as proteins Sp100 and BLM, either protein knocking out does not result in fewer PML nuclear bodies (Zhong et al., 1999; Ishov et al., 1999). Similarly, P-bodies also recruit some RBPs that are not essential for P-body assembly, partially via scaffolding interactions between RBPs and RNA (Buchan and Parker, 2009). Furthermore, MLCs likely have multiple scaffolds with contributions from different types of multivalent interactions, as well as numerous clients possessing multiple categories of low-valency elements that can each interact with scaffolds. In conclusion, the assembly of condensates was regulated by the valency, concentration, and molar ratio of a small number of scaffold molecules (Banani et al., 2016).

The latest data suggest that multivalent interactions of folded domains, IDRs, and RNA described above can synergistically promote the formation of MLCs in cells. For SG formation, LLPS is driven by the collective interaction network that breaks the saturation concentration threshold. The core network consists of 36 proteins and associated mRNAs, but protein-RNA interactions driving LLPS are distributed unevenly, with some nodes more essential than others (Yang et al., 2020). Specifically, RBP G3BP1 is the central node, and dimerization via its NTF2L domain could increase the valency and ability of RNA-binding to mediate LLPS. However, no specific consensus sequence is required for G3BP1-RNA interaction. In the author’s opinion, RNA-binding by the RRM domain in G3BP1 is produced by competition between intramolecular and intermolecular interactions related to IDRs: upon increased RNA concentration, RNA displaces the acidic IDR1 to bind the basic IDR3, which alters the conformation of G3BP1 homodimer from compact to expanded, initiating LLPS. Thus, multivalency for RNA-binding contributes to RNA-dependent LLPS; intermolecular RNA-RNA interactions are dispensable here, but they can lower the components required for LLPS. In addition to the intrinsic properties of G3BP1 and RNA, the threshold for LLPS is regulated by positive or negative synergies of other proteins, such as caprin-1 and USP10, which lower and heighten the saturation concentration of G3BP1 and RNA required for SGs assembly, respectively (Yang et al., 2020).

## 3 RNA contributions to the formation of MLCs

**FIGURE 3:**
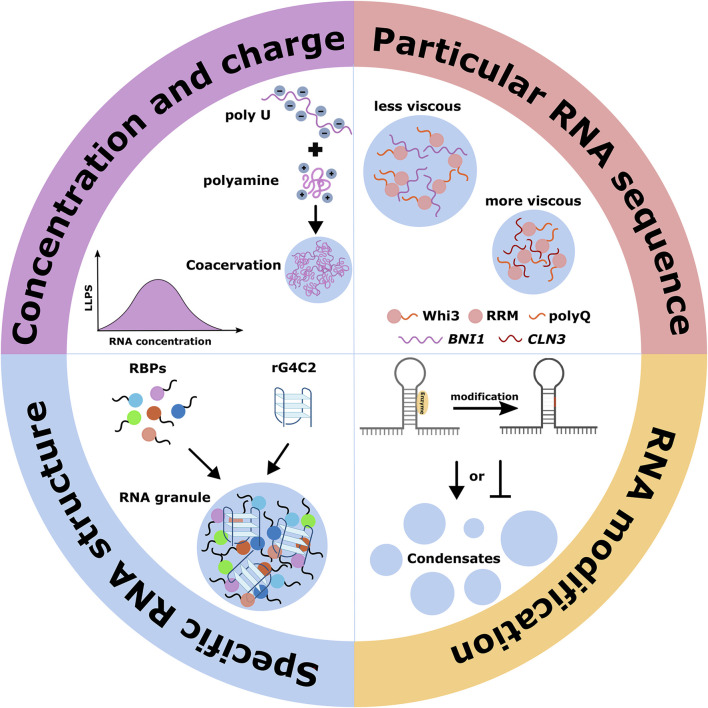
RNA contributions in the formation of phase-separated MLCs. The compartmentalization process is affected by different properties of RNAs, including the concentration, charge, particular RNA sequence, specific RNA structure, and RNA modification.

As research continues, profound roles for RNA in regulating LLPS processes have been revealed in recent years. RNA itself or RNA-binding to other molecules potently affects phase behavior, controlling the formation, composition, material properties, maintenance, and even efficient dissolution of condensates (Drino and Schaefer, 2018). Many nuclear MLCs, such as the nucleolus, Cajal body, and paraspeckle, form in coordination with the transcription of specific RNAs. In addition, upon transcriptional inhibition after actinomycin D treatment, these MLCs are rapidly disassembled alongside their protein components to separate them into unique structures (Shav-Tal et al., 2005). For instance, the nucleolus is a typical MLC dedicated mainly to the biogenesis of ribosomes, a process covering rRNA transcription, pre-rRNA processing, and mature rRNA-ribosomal protein assembly (Shan et al., 2023). Although nucleolus forms around the rDNA repeat, it has been demonstrated that the accumulation of rRNA transcripts, rather than the presence of rDNA, drives the formation of the nucleolus (Falahati et al., 2016). Cajal bodies are another nuclear substructure implicated in small nuclear RNPs (snRNPs) biogenesis, eventually forming the spliceosomes. It is demonstrated that small nuclear RNAs (snRNAs) and the IDR-containing protein coilin are enriched in Cajal bodies, and snRNAs directly interact with and induce coilin condensation, making the Cajal bodies phase separation. In addition to snRNAs, small Cajal body-specific RNAs (scaRNAs) and all small nucleolar RNAs (snoRNAs) were identified as coilin interactors, further highlighting RNA contributions in Cajal body formation (Machyna et al., 2014). Many discrete nuclear compartments produce the “speckled” appearance, such as nuclear speckles, paraspeckles, PML nuclear bodies, and nuclear stress bodies, which are distinguished by the factors they contain and are involved in the consecutive steps of mRNA biogenesis in the nucleus (Hirose et al., 2023). Especially the notable feature of paraspeckles is that lncRNA NEAT1 is highly concentrated and controls paraspeckle formation and maintenance as a scaffold, distinct from other nuclear RNAs that localize to but are non-essential for specific structures. The researchers speculated that it might be attributed to the 4 kb complex molecular structure of NEAT1 RNA containing many self-complementary sequences, which could form intra/inter-molecular hybrids and even the lattice to build larger-scale architectural complexes (Clemson et al., 2009). Similarly, the highly repetitive satellite III (HSATIII) lncRNA plays an architectural role in forming nuclear stress bodies in primates upon thermal stress exposure by recruiting various RBPs (Ninomiya et al., 2020). RNAs play roles in MLC assembly, disassembly, and size/shape modulation, and different properties of RNAs such as concentration, charge, particular RNA sequence, specific RNA structure, and RNA modification all contribute to these processes (Figure 3).

### 3.1 Concentration and charge

First, local RNA concentration could impact the material properties of assembled MLCs. Under normal conditions, prion-like RBPs such as TDP43 and FUS are primarily soluble in the nucleus but whose mislocalization to the cytoplasm causes solid-like aggregate formation. The phenomenon can be explained by the effects of overall RNA/protein ratios on the phase behavior of these prion-like proteins: high nuclear RNA concentrations prevent phase separation, and low cytoplasmic RNA concentrations promote it (Maharana et al., 2018). RNAs facilitate the LLPS of proteins by reducing the protein concentration required for phase separation (Drino and Schaefer, 2018). With the concentration of RNA monotonically increasing, condensates initially assemble via complex coacervation but subsequently disassembly because of the high concentration of local negative charge provided by accumulated RNA, which can regulate the electrostatic interactions with other molecules during LLPS (Banerjee et al., 2017). In addition, the general anionic properties of RNAs could contribute to complex condensation, which has been observed in an RNA-cation system: the addition of the polycytidylic acid (polyU) to polyamines in solution resulting in condensation (Figure 3). Moreover, the coacervation formed only above the melting transition temperature (Tm), breaking the weak secondary structure of polyU at low temperatures (Aumiller et al., 2016).

### 3.2 Particular RNA sequence

RNAs also embed fundamental features such as particular sequences to determine their various roles, which almost always function with their protein partners. The molecular mechanisms in which individual RNA-recognition domains recognize specific RNAs have been discussed in detail (Maris et al., 2005; Chang and Ramos, 2005; Auweter et al., 2006; Lunde et al., 2007). It has been suggested that RNA sequences influence condensate biophysical properties such as the viscosity of condensates, their tendency to fuse, and the exchange rates of components with a bulk solution. For instance, Whi3, an RBP with a long polyQ tract adjacent to an RRM, is essential for the spatial patterning CLN3 and BNI1 mRNA in the cytosol, which regulate nuclear division and polarity, respectively. RNA-binding through the RRM domain is critical for Whi3 phase separation. Different target mRNAs drive Whi3 assembly into condensates with distinct biophysical properties: less viscous Whi3 condensates assembled with BNI1 mRNA are identified to establish polarity sites at new branch sites, and more dense Whi3 condensates assembled with CLN3 mRNA are adjacent to nuclei to control the timing of nuclear division (Zhang et al., 2015; Langdon et al., 2018; Lee et al., 2015) (Figure 3). Recently, a class of lncRNAs functioning as scaffolds during nuclear body formation has been defined as architectural RNAs (arcRNAs), whose transcription is coupled with the co-transcriptional binding of some RBPs to specific RNA elements embedded in the arcRNAs (Chujo et al., 2016). A high transcriptional level of arcRNAs facilitates the assembly of the nuclear body, in which some of these proteins could be adsorbed to arcRNAs and maintain their steady-state level, and others facilitate protein-protein interactions via conformational changes resulting from binding to arcRNAs. As in the case of paraspeckles assembly, SFPQ, NONO, and RBM14 are required to maintain the paraspeckle structure by protecting the NEAT1 lncRNA from degradation, and FUS binding with NEAT1 lncRNA promotes further intra/inter-molecular protein-protein interactions of FUS for the formation of paraspeckle structures (Chujo et al., 2016).

### 3.3 Specific RNA structure and RNA modification

Indeed, most protein-RNA interactions are driven by the affinity of RBP to specific motifs, including RNA sequence and structure. Most RBPs require the presence of RNA-binding domains to recognize sites composed of 3-8 nucleotides efficiently and can withstand a high degree of sequence variation in these binding sites (Mitchell and Parker, 2014). However, numerous studies show that the presence of binding sites in expressed transcripts does not guarantee bound by their cognate RBP *in vivo* and *in vitro*, in which local RNA structures play a decisive role in the widespread inhibition of RBP binding (Taliaferro et al., 2016). Based on the WC and non-WC base pairs and their interaction, RNAs form various structural elements such as stems, loops, hairpins, pseudoknots, helical regions, and G-quadruplexes, which determine the RNA three-dimensional topologies in hinge regions (Miao and Westhof, 2017). Likely, the extent to which the RNA structure encloses the RNA sequence determines whether an RNA is more likely to form favorable interactions with a protein than others (Jankowsky and Harris, 2015; Singh and Valcarcel, 2005; Qi et al., 2021). These are also applied to LLPS-driven MLCs: RNAs, especially those containing repeat sequences, likely influence the LLPS and MLCs dynamics by facilitating intra/inter-molecular assembly and forming higher-order structures.

A good example is the hexanucleotide GGGGCC RNA (rG4C2) transcribed from the C9ORF72 locus, which promotes LLPS of RNA granule proteins *in vitro* and cells (Fay et al., 2017). The ability of rG4C2 to mediate phase separation is dependent on its G-quadruplex structure that is formed by stacks of four guanosine residues hydrogen bonded by Hoogsteen base pairs, coordinated by specific metal cations, which creates a local microenvironment that drives RNA granule formation (Figure 3). Moreover, rG4C2-mediated condensation is fast, reversible, temperature- and concentration-dependent, and highly responsive to metal ionic/polycationic conditions (Fay et al., 2017). Accordingly, the concentration and charge of particular nucleotides, RNA sequence, and RNA structure and protein-binding are inherent features of biomolecular condensates formation; in addition, RNA modifications occurring at specific positions could influence these characteristics by modulating charge, base stacking, steric hindrance, and hydrophobicity as well as pairing potential of bases (Drino and Schaefer, 2018; Roden and Gladfelter, 2021).

## 4 lncRNA and LLPS

Mounting evidence suggests that changes in RNA levels or RNA elements cause aberrant phase separations, especially lncRNAs, whose sequences range from 200 bases to tens of kilobases, are more capable of providing binding sites for diverse RNAs and protein interaction in both the nucleus and cytoplasm. Various other features of lncRNAs, such as nucleotide composition, structures, and modifications, also facilitate compartmentalization (Guo et al., 2021). Moreover, lncRNAs serve as buffers to decrease the viscosity of protein components and promote the diffusion of protein components. In phase separation condensates, lncRNAs also serve as enhancer RNAs(eRNAs), participating in diverse cellular functions, including chromatin modification, transcription, splicing, mRNA decay, translation, protein transport, and assembly. Here, we briefly discussed some of the lncRNAs reported as the mainstay in LLPS.

### 4.1 Genome architecture maintenance

It has been shown that some nuclear matrix (NM)-associated proteins could organize 3D chromatin structures by interacting with non-coding RNAs (Fan et al., 2018; Nozawa et al., 2017; Hasegawa et al., 2010; Hacisuleyman et al., 2014). Recent studies further suggested that 3D genome architectures may be organized following the principle of LLPS, in which many RBPs cooperate with RNAs, including lncRNAs, to facilitate these processes. For example, via its R/G-rich region, an NM-associated protein SAFB interacts with lncRNA MajSAT to stabilize heterochromatin foci by promoting phase separation (Huo et al., 2020). Loss of MajSAT results in dissociation of the SAFB condensate, thereby debilitating heterochromatin foci labeled by H3K9me3 staining. Similarly, lncRNA Xist triggers phase separation to mediate X-chromosome inactivation (XCI). Early stages of XCI initiation are mediated through the direct recruitment of repressive chromatin marks by Xist, resulting in early gene silencing. Subsequently, Xist drives phase separation by enriching IDR-containing proteins to reinforce the silencing process (Pandya-Jones et al., 2020; Cerase et al., 2019). NcRNA activated by DNA damage (NORAD), a highly abundant and conserved cytoplasmic lncRNA, is also needed for mammal genomic stability through negative Pumilio (PUM) protein regulation. The PUM proteins are post-transcriptional repressors that target mRNAs harboring Pumilio response elements (PREs) (UGUANAUA) to promote mRNA decay and repress translation (Goldstrohm et al., 2018). Because of 18 PRE content, NORAD can out-compete thousands of other PUM-binding transcripts to inhibit the activity of PUM proteins by assembling them into phase-separated condensates, termed NP bodies. NORAD-driven PUM phase separation disturbance leads to PUM protein hyperactivity and abnormal mitosis, which can be rescued by synthetic RNAs that induce the NP body formation (Elguindy and Mendell, 2021). Furthermore, lncRNA-mediated LLPS is also involved in DNA damage response (DDR) to maintain genome integrity. Upon DNA double-strand breaks (DSBs), MRE11/RAD50/NBS1(MRN) complex and Pol II stabilize each other at exposed DNA ends to trigger damage-induced lncRNAs (dilncRNAs) synthesis, which can further drive phase separation of DDR factors, such as 53BP1, to generate signaling and repair reactions (Sharma et al., 2021; Pessina et al., 2019). Interestingly, lncRNA can also be processed into shorter DDR RNA (DDRNA) through the DROSHA and DICER processes, and these two RNA pairing allow them to bind together into 53BP1 and accumulate at DDR focus; however, whether and how DDRNA intervening condensate formation is still unknown (Michelini et al., 2017).

### 4.2 Gene transcription regulation

The transcription of most genes in eukaryotes is mainly divided into transcription initiation, elongation, splicing, and termination steps, each involves the interaction of RNA polymerase II (Pol II) with the corresponding transcription initiation factor, elongation factor, splicing factor or termination factor, respectively (Core and Adelman, 2019; Cramer, 2019; Chen et al., 2018). Recent reports show that IDRs-mediated LLPS selectively partition different transcriptional programs into specific transcriptional condensates to control these gene activation steps (Stortz et al., 2024; Henninger et al., 2021; Lyons et al., 2023). A new view of eukaryotic gene transcription initiation is that forming phase-separated transcriptional condensates at super-enhancers creates high local concentrations of sequence-specific TFs, coactivators such as mediators, and Pol II to activate gene expression. By their IDRs (e.g., from TF activation domain, mediator, and Pol II C-terminal domain (CTD)), these proteins exhibit affinity interactions: activation domains of TFs can interact with enhancer-associated mediators to form mediator condensates, and mediators can provide a “scaffold” for “client” Pol II molecules at multiple gene promoters (Palacio and Taatjes, 2022; Mann and Notani, 2023). Moreover, the property of transcriptional condensates is regulated by biochemical reactions to complete efficient and controllable transcription. For instance, Pol II CTD phosphorylation drives the shuttle from initiation condensates to elongation condensates, and hyperphosphorylation leads to splicing condensate replacement. Once Pol Ⅱ reaches the end of the gene, hypophosphorylated CTD is recycled by the initiation condensate (Lu et al., 2018; Peng et al., 2020; Rawat and Sawarkar, 2023; Guo et al., 2019).

Also, Pol Ⅱ initiates bidirectional transcription from enhancer elements to produce eRNAs, which generally correlate with enhancer activation and contribute to condensate formation and dissolution (Henninger et al., 2021; Nair et al., 2019). A recent study provides unequivocal evidence that N6-methyladenosine (m6A)-modified eRNA recruits the nuclear m6A reader YTHDC1 to assemble liquid-like condensates, which further facilitates BRD4 coactivator condensate formation and, therefore, enhancer stimulation and gene transcriptional A activation. The arginine residues of YTHDC1 C-terminus IDR are indispensable for its condensate assembly and for expanding BRD4 condensates, and depletion of YTHDC1 dose does not affect eRNA stability but inhibits BRD4 condensate formation and its recruitment to enhancers, resulting in gene inactivation (Lee et al., 2021). BRD4, a famous BET family member, binds to acetylated histones via bromodomains (BDs) to affect transcriptional elongation at enhancers and promoters (Sabari et al., 2018). In addition, BRD4 and other family members were also demonstrated to directly interact with eRNAs through BD modules, which further enhance BRD4 binding to acetylated histones (Rahnamoun et al., 2018). However, whether their interaction forms BRD4 condensate is presently unknown. Similarly, the other histone acetylation reader, BRD3, is recruited to H3K18ac to form phase-separated condensates to control gene expression in endoderm differentiation, which is dependent on the interaction between lncRNA DIGIT and BRD3 bromodomains (Daneshvar et al., 2020).

## 5 Skeletal myogenesis and LLPS

**FIGURE 4:**
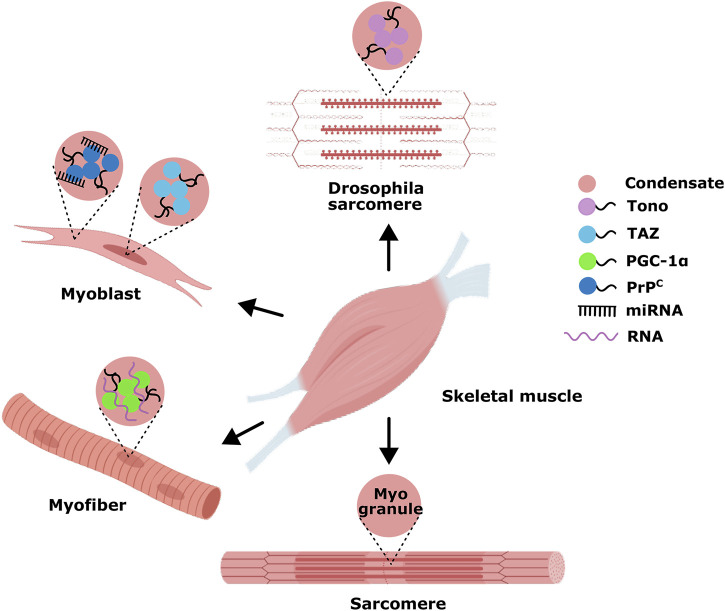
Examples of phase-separated MLCs in skeletal myogenesis. Only a few studies show that phase-separated MLCs in skeletal muscle regulate gene transcription during myogenesis.

It is becoming increasingly apparent that the assembly of condensates through LLPS is an essential characteristic of spatio-temporal control of various biomolecular processes. For skeletal myogenesis, LLPS-relevant research mainly involves the aggregation of some RBP to regulate muscle development (Figure 4). For example, TDP-43 is an essential RBP for skeletal myogenesis that unexpectedly forms cytoplasmic condensates, termed myo-granules(MGs), upon injury and are cleared as the myofibers mature (Vogler et al., 2018). It has been demonstrated that MGs help orchestrate the organization of sarcomeres, the contractile unit of skeletal muscle. As sarcomeric mRNAs are extremely large, whose packaging and transporting to sites of newly forming sarcomeres may be solved by MGs, serving a similar role as mRNP granules in neurons (Corbet et al., 2021; Alami et al., 2014). In 2024, Julia Zibold et al. observed the new missense G376V variant led to the formation of cytoplasmic TDP-43 condensates in patient-derived myoblasts and induced abnormal mRNA splicing in patient muscle tissue (Zibold et al., 2024). The missense mutation in TDP-43 is located within its LCD. Compared to the large, spherical condensates formed by wild-type TDP-43, the G376V variant exhibits an increased propensity for TDP-43 aggregation in myoblasts and myotubes, forming more amorphous condensates driven by aberrant phase separation. This suggests that the mutant TDP-43 resides in an unstable thermodynamic state, which may disrupt the splicing of mRNAs encoding muscle-specific structural and contraction-associated proteins (Zibold et al., 2024). Though numerous reports showed that TDP43 undergoes LLPS mediated by LCD and even converts into a gel or solid phase both *in vitro* and in cells, the phase separation properties of TDP43 are still unclear in MGs formation (Yu et al., 2021; Li et al., 2018a; Li et al., 2018b). A recent study shows that the BTB Zn-finger protein Tono is also expressed during sarcomere maturation and displays robust phase separation properties in *Drosophila* indirect flight muscle nuclei. Specifically, in response to external mechanical pressure, Tono forms liquid condensates utilizing its BTB domain in muscle cell nuclei and thus may adjust the transcriptional switch of the sarcomere according to its internal status (Zhang et al., 2023). TAZ inhibits myogenic differentiation by antagonizing a pro-myogenic transcription complex as the Hippo kinase pathway’s target nuclear regulatory protein. Upon phosphorylation by Hippo activation, TAZ is sequestered to the cytoplasm to reset the myogenic differentiation program (Watt et al., 2018; Sun et al., 2017). Emerging research highlights that TAZ displays robust phase separation properties in myonuclear speckles: through dynamic assembly into nuclear condensates, TAZ may concentrate myogenic nuclear machinery comprising Smad7 and β-catenin to repress skeletal myogenesis (Tripathi et al., 2022; Tripathi et al., 2019). Until now, there have been only sporadic reports about RNA-mediated LLPS in skeletal myogenesis. For instance, there is direct evidence that LLPS assembled active transcriptional condensates in human and mouse skeletal muscle, in which the solid transcriptional coactivator PGC-1α interacted with RNAs and multivalent proteins via its highly conserved CTD to compartmentalize active transcription (Perez-Schindler et al., 2021). Furthermore, miRNA-mediated LLPS *in vitro* and within cells was observed in human and mouse skeletal muscle cells under pathological conditions. In 2023, Tao et al. first observed excess accumulation and co-localization of miR-214-3p with PrPC in skeletal muscle samples of skin disease patients, revealing miRNA’s mechanisms for the LLPS of PrPC, muscle cell self-depletion and differentiation regulation. The cellular prion protein PrP^C^ selectively recruits specific miRNAs such as miR-214-3p into phase-separated condensate in myoblasts, which in turn enhances the LLPS of PrP^C^, resulting in E3 ubiquitin ligase ATG5-related autophagy inhibition, and blocks myoblast differentiation (Tao et al., 2023).

## 6 Conclusions and perspectives

It has become increasingly clear that multiple MLCs dynamically coexist in the nucleus or cytoplasm, and they sometimes share many components and even contact or fuse to form new MLC-like foci with unique physicochemical properties. Hence, it is essential to understand how the sequence-encoded information drives the coexistence of complex multiphases and the biological functions of these content-rich MLCs. This review discusses the molecular mechanism of MLC formation driven by LLPS and how their features affect various cellular processes, focusing on RNAs, especially lncRNAs. MLCs mainly consist of specific proteins and RNAs, and the multivalent interaction (protein-protein, protein-RNA, and RNA-RNA) is dictated by their respective inherent affinity, concentration, protein/RNA modification, competition relation, and surrounding biophysical conditions. Phase-separated compartments could cluster together a protein/RNA and its potential interaction partners while excluding others to maintain the specific functions. On the other hand, condensates create a high local concentration of resident molecules to accelerate reactions inside the compartments. Nevertheless, we have yet to learn about this process so far. For example, what are the exact biological functions of phase-separated condensates (e.g., transcriptional control, ribonucleoprotein processing)? What are the differences between scaffold and client molecules and how their sequence and structural properties dictate phase behavior? How do external factors (e.g., mechanical forces, metabolic signals, or stress) specifically alter MLC dynamics? It needs to be explored.

Finally, we described a few emerging research topics of phase-separated MLCs in skeletal myogenesis, and these dynamic, biomolecular assemblies play pivotal roles in orchestrating spatiotemporal regulation, concentrating key factors (e.g., RNAs and RBPs), and facilitating efficient molecular interactions. However, LLPS-driven MLCs in skeletal myogenesis is still in its infancy, future research should prioritize tracking MLC formation, fusion, and dissolution in real time during muscle development, as well as investigating MLC dysregulation in muscle disorders (e.g., atrophy, myopathies) and aging. Moreover, considering the presence of lncRNAs makes the phase-separated condensate more dynamic, which enhances the compartmentalization process; one of the subsequent investigations in skeletal myogenesis is to understand the potential regulation of lncRNAs that are likely to interact with RBPs to assemble muscle MLCs and the complex biology and their functional roles behind. In conclusion, the emergence of LLPS-driven MLCs has undoubtedly revolutionized our understanding of all biological activities. Basic research in this field will provide critical insights into skeletal muscle developmental biology.

## Glossary

MLC: Membrane-less condensate
LLPS: Liquid-liquid phase separation
IDR: Intrinsically disordered region
MLO: Membrane-less organelle
P-body: Cytosolic processing body
SG: Stress granule
N-WASP: Neural Wiskott-Aldrich Syndrome protein
pTyr: phosphotyrosine
PRM: Proline-rich motif
PSD: Postsynaptic density
NPM1: Nucleolar protein 1
UBA: Ubiquitin-associated
R-motif: Arginine-rich linear motif
LCD: Low-complexity domain
PLD: Prion-like domain
mRNP: messenger ribonucleoprotein
RGG: Arg/GlySYGQSer/Tyr/Gly/Gln
SYGQ: Ser/Tyr/Gly/Gln
Q/N: Gln/Asn
RBP: RNA-binding protein
RBD: RNA-binding domain
WC: Watson-Crick
PML: Promyelocytic leukemia protein
snRNP: small nuclear RNP
snRNA: small nuclear RNA
scaRNA: cajal body-specific RNA
snoRNA: small nucleolar RNA
HSATIII: Highly repetitive satellite III
polyU: polycytidylic acid
arcRNA: architectural RNA
rG4C2: hexanucleotide GGGGCC RNA
eRNA: enhancer RNA
NM: Nuclear matrix
XCI: X-chromosome inactivation
NORAD: ncRNA activated by DNA damage
PRE: Pumilio response element
DDR: DNA damage response
DSB: DNA double-strand break
MRN: MRE11/RAD50/NBS1
dilncRNA: damage-induced lncRNA
CTD: C-terminal domain
m6A: N6-methyladenosine
BD: Bromodomain
MG: Myo-granule
