# Supplementary figures and images for: Liquid-liquid phase separation of membrane-less condensates: from biogenesis to function
Source: Front Cell Dev Biol. 2025 May 14;13:1600430. doi: 10.3389/fcell.2025.1600430 (PMC12116561; doi:10.3389/fcell.2025.1600430)

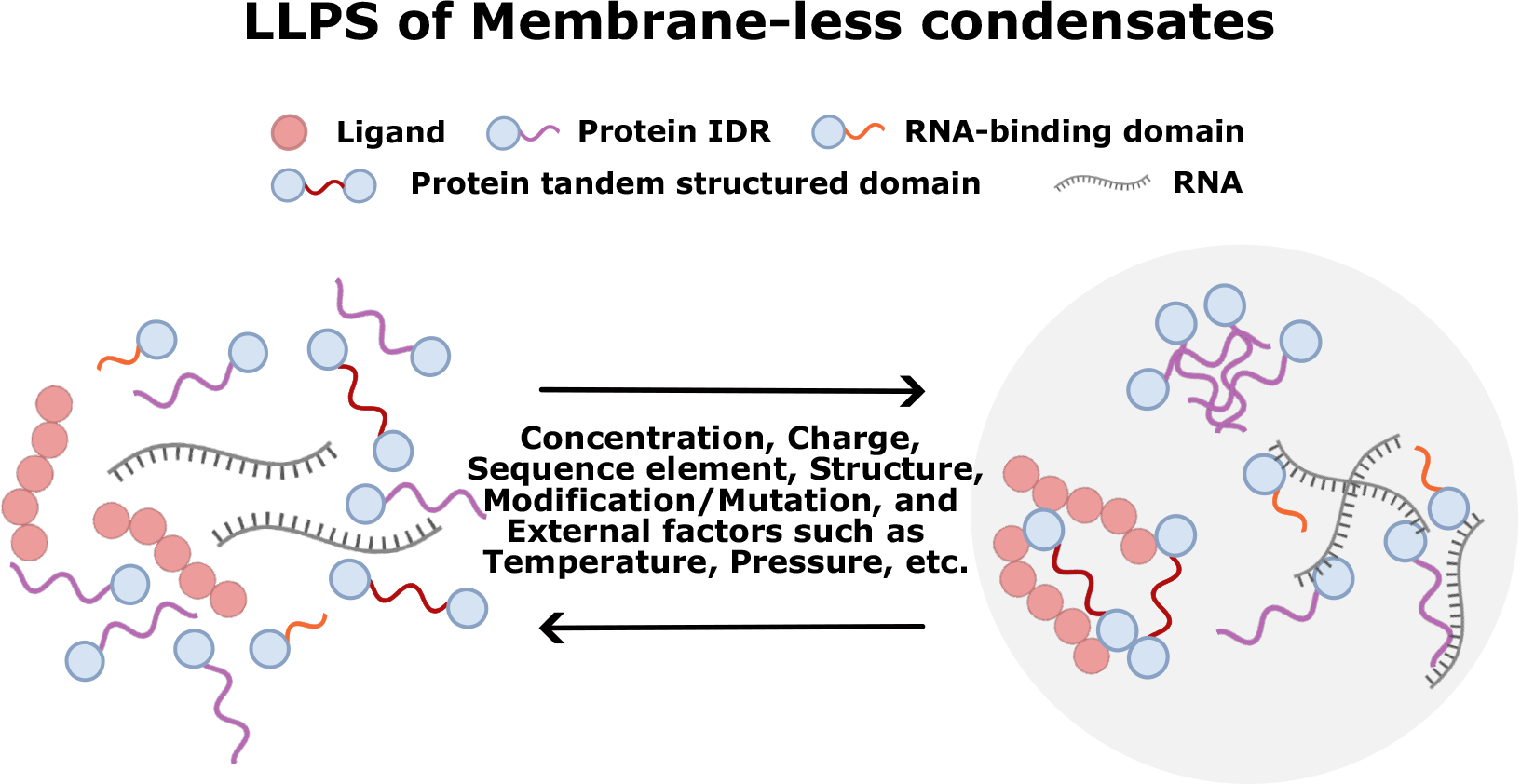

Supplement: Supplementary file 1 [file Image1.tif]
